# Single-stage concomitant extended thymectomy for myasthenia gravis and enucleation of a left recurrent laryngeal nerve schwannoma via median sternotomy

**DOI:** 10.1186/s44215-026-00248-3

**Published:** 2026-03-10

**Authors:** Go Kamimura, Koki Maeda, Masaya Aoki, Satomi Imamura, Shoichiro Morizono, Yuto Nonaka, Aya Takeda, Toshiyuki Nagata, Kazuhiro Ueda

**Affiliations:** Department of General Thoracic Surgery, Graduate School of Medical and Dental Sciences Kagoshima University, 8-35-1, Sakuragaoka, Kagoshima City, Kagoshima Prefecture 892-8520 Japan

**Keywords:** Myasthenia gravis, Extended thymectomy, Recurrent laryngeal nerve, Schwannoma, Median sternotomy

## Abstract

**Background:**

Myasthenia gravis (MG) without thymoma is often treated with extended thymectomy to improve symptom control and reduce immunotherapy requirements. Schwannoma of the recurrent laryngeal nerve (RLN) is rare, and surgical resection is usually curative. However, operative manipulation carries a risk of temporary or permanent vocal fold paralysis. When these conditions coexist, choosing staged versus single-stage surgery is non-trivial. A synchronous operation can consolidate perioperative care and recovery but demands careful planning to prevent respiratory complications, particularly if RLN palsy or myasthenic crisis occur.

**Case presentation:**

A 65-year-old woman with generalized, thymoma-negative MG (acetylcholine-receptor antibody positive) had no hoarseness or dysphagia. Flexible laryngoscopy confirmed mobile vocal folds bilaterally. Chest radiography showed mediastinal widening without diaphragmatic paralysis. Contrast-enhanced CT and MRI revealed a right anterior mediastinal cystic lesion and a solid mass in the left tracheoesophageal groove abutting the RLN. ^18F-fluorodeoxyglucose positron emission tomography demonstrated uptake in the solid lesion (SUV_max 4.89). Transesophageal endoscopic ultrasound–guided fine-needle aspiration yielded spindle cells compatible with schwannoma, and the mass was considered RLN-derived. A single-stage median sternotomy was undertaken. Extended thymectomy was performed with bilateral phrenic exposure and en bloc removal of thymic/perithymic fat, followed by nerve-sparing enucleation of the left RLN tumor through a longitudinal epineurial window and meticulous intracapsular dissection, preserving macroscopic neural continuity. The postoperative intensive care unit course was uneventful: no dyspnea or dysphagia, normal voice, and laryngoscopy confirmed intact vocal fold mobility. A chest radiograph on postoperative day 5 was unremarkable, and the patient was discharged on day 7. Histopathology showed alternating Antoni A/B areas with virtually no mitoses; tumor cells were strongly S-100–positive, confirming schwannoma.

**Conclusions:**

In carefully selected patients with stable MG and no preoperative vocal fold dysfunction, single-stage median sternotomy enables safe concomitant extended thymectomy and RLN schwannoma enucleation. Success hinges on nerve-sparing technique and proactive perioperative planning for airway protection and potential MG crisis.

## Background

Myasthenia gravis (MG) without thymoma is commonly managed with extended thymectomy in appropriately selected patients, aiming to improve long-term symptom control and reduce immunotherapy requirements [1]. On the other hand, recurrent laryngeal nerve (RLN) schwannoma [2] is a rare benign peripheral nerve sheath tumor that typically arises in the left tracheoesophageal groove; while surgical excision is generally curative, manipulation of the RLN carries a risk of temporary or permanent vocal fold paralysis. When the two diseases occur together, the choice between single-stage surgery and a planned two-stage approach requires careful consideration. A synchronous operation may shorten overall recovery and consolidate perioperative care, yet it poses unique challenges: potential postoperative airway compromise if RLN palsy [3] occurs; aspiration risk with dysphonia; and, in MG, the possibility of respiratory failure precipitating myasthenic crisis [4]. These risks necessitate meticulous planning of anesthesia and postoperative surveillance, judicious perioperative immunomodulation, and early laryngoscopic assessment when indicated. Here, we describe a thymoma-negative MG patient who underwent extended thymectomy with concomitant enucleation of a left RLN schwannoma via median sternotomy in a single session, achieving uneventful vocal fold function and favorable MG control.

## Case presentation

A 65-year-old female patient with generalized MG was referred for surgical management. She reported fluctuating fatigability of the ocular and bulbar musculature but had no hoarseness or dysphagia on presentation. Physical examination revealed no stridor; bedside voice assessment was normal, and flexible laryngoscopy demonstrated bilaterally mobile vocal folds. Serum acetylcholine receptor–binding antibodies were positive, and the clinical picture was consistent with thymoma-negative, generalized MG. Given the stability of symptoms and pulmonary function, the neuromuscular team judged that preoperative pharmacologic control was unnecessary. General anesthesia was induced and maintained with standard total intravenous anesthesia (TIVA), and double-lumen endotracheal intubation was used for ventilation.

**Fig. 1:**
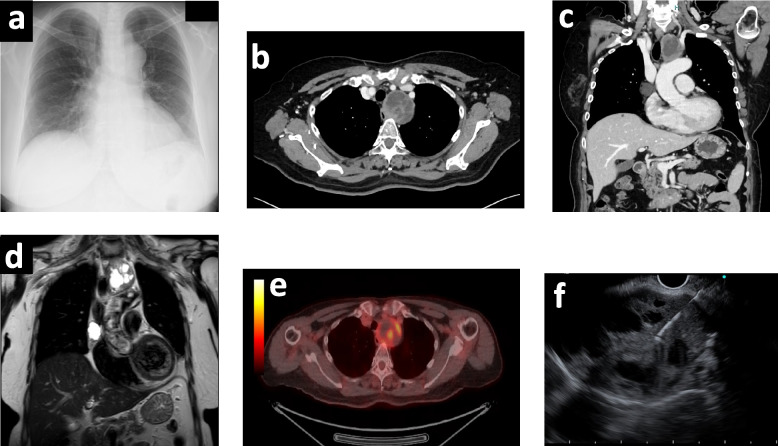
Preoperative imaging. **a** Upright chest radiograph showing mediastinal widening without diaphragmatic paralysis. **b**–**c** Contrast-enhanced CT: solid, well-circumscribed mass in the left tracheoesophageal groove and a right anterior mediastinal cystic lesion. **d** Contrast-enhanced MRI: encapsulated solid tumor with multiloculated cystic components and a separate mediastinal cyst. **e** 18F-FDG PET: uptake in the solid lesion (SUV_max 4.89). **f** Transesophageal EUS-guided FNA: spindle cells compatible with schwannoma

A standing chest X-ray demonstrated mediastinal enlargement with no signs of diaphragmatic paralysis (Fig. 1a). Contrast-enhanced chest computed tomography identified two distinct mediastinal findings: (i) a solid, well-circumscribed mass along the left tracheoesophageal groove, closely related to the course of the left RLN (Fig. 1b, c), and (ii) a right-sided, well-demarcated lesion in the anterior mediastinum with cystic components (Fig. 1c). Contrast-enhanced chest magnetic resonance imaging (MRI) demonstrated a mediastinal cystic lesion and a solid, encapsulated tumor with multiloculated cystic components (Fig. 1d). On 18F-fluorodeoxyglucose positron emission tomography (18F-FDG PET), the solid tumor showed increased uptake with a maximum standardized uptake value (SUVmax) of 4.89 (Fig. 1e). To establish the nature of the paratracheal mass, transesophageal endoscopic ultrasound–guided fine-needle aspiration was performed, yielding spindle cells compatible with a schwannoma; in view of the anatomic relationship, the lesion was considered to be left RLN-derived (Fig. 1f).

**Fig. 2:**
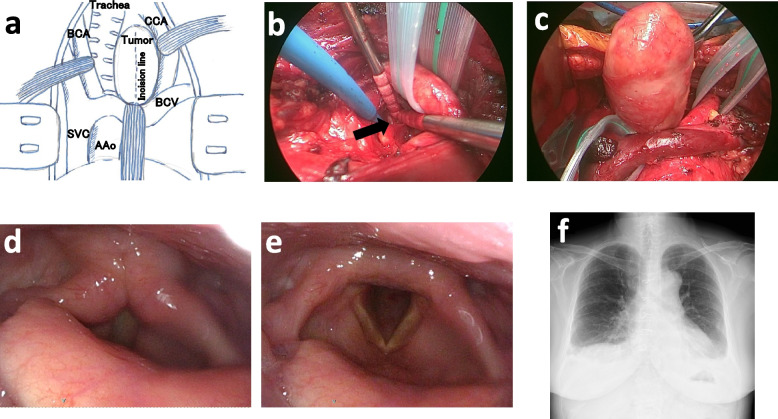
Operative and postoperative findings. **a** Schema of the surgical field; Wide Penrose tapes were placed around the brachiocephalic vein (BCV), common carotid artery (CCA), and brachiocephalic artery (BCA) to retract the great vessels and secure a clear operative field for tumor identification. AAO, Ascending aorta; SVC, Superior vena cava. **b** Identification and preservation of RLN fascicles (arrow). **c** Enucleation of the encapsulated mass with preserved macroscopic neural continuity. **d**–**e** Postoperative laryngoscopy: intact bilateral vocal fold mobility. **f** Upright chest radiograph on postoperative day 5 without abnormalities

After multidisciplinary discussion, the patient proceeded to a single-stage operation via median sternotomy. An extended thymectomy was carried out with exposure of both phrenic nerves and en bloc removal of thymic and perithymic adipose tissue from the diaphragm to the lower neck. The left tracheoesophageal groove was then explored. Intraoperative nerve monitoring equipment was prepared and kept on standby for use as needed. After taping the brachiocephalic vein, common carotid artery, and brachiocephalic artery with wide Penrose drains to secure exposure (Fig. 2a), the tumor was identified. Nerve fascicles were encountered and preserved (Fig. 2b), and the lesion was dissected without the use of electrocautery using gentle digital blunt dissection. Using meticulous intracapsular dissection, the encapsulated mass was enucleated while preserving gross neural continuity (Fig. 2c). There was no need for nerve resection or reconstruction. Operative blood loss was minimal, and the procedure was completed with hemodynamic stability.

**Fig. 3:**
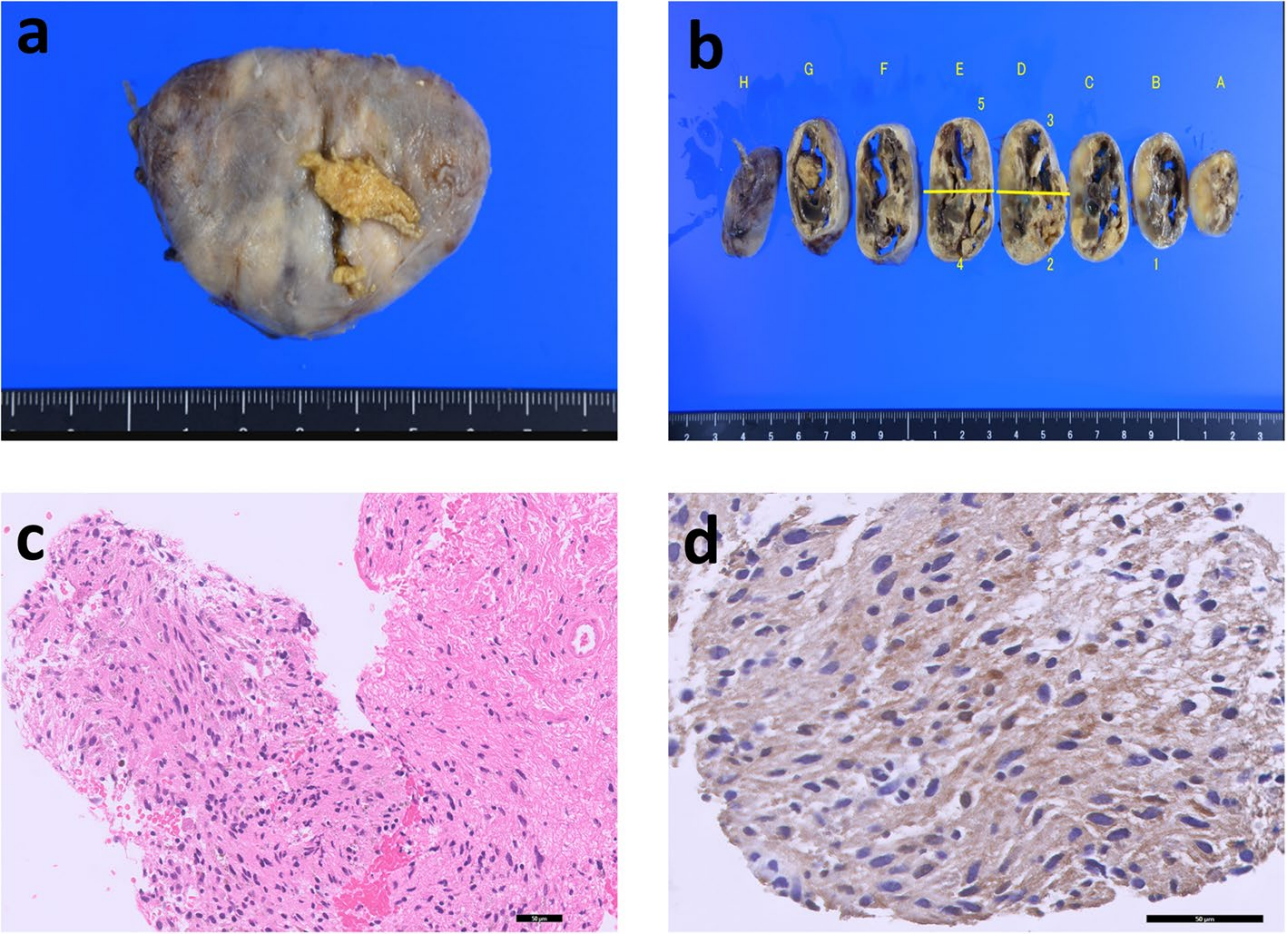
Gross and pathology. **a**–**b** Resected tumor and cut surface. **c** H&E: spindle cells in sweeping fascicles with alternating Antoni type A/B areas. **d** Immunohistochemistry: diffuse S-100 positivity confirming schwannoma

Postoperatively, the patient was extubated in the operating room and managed in the intensive care unit (ICU) for close monitoring of potential myasthenic crisis and airway compromise. Her course was uneventful: there was no dyspnea or dysphagia, voice quality remained normal, and laryngoscopy confirmed intact vocal fold mobility (Fig. 2d, e). The standing chest X-ray on postoperative day 5 showed no abnormalities (Fig. 2f). On hematoxylin–eosin staining, the tumor was composed of spindle cells arranged in sweeping, fascicular patterns with virtually no mitotic figures (Fig. 3c). Alternating hypercellular areas consistent with Antoni type A [5] architecture and hypocellular, loosely textured Antoni type B [5] regions were observed. The resected tumor and cut surface are shown (Fig. 3a, b). Immunohistochemically, the tumor cells were strongly positive for S-100 protein, supporting a diagnosis of schwannoma (Fig. 3d). The patient was discharged home on postoperative day 7 with standard MG follow-up arranged.

## Discussion

Extended thymectomy is a well-established option for thymoma-negative MG in appropriately selected patients, aiming to reduce symptom burden and long-term immunotherapy requirements. RLN schwannoma is an uncommon benign tumor that arises from Schwann cells along the tracheoesophageal groove. Although simple excision is curative in most cases, the principal surgical risk is temporary or permanent vocal fold paralysis with attendant airway and aspiration concerns. The coexistence of these two conditions poses a management dilemma: whether to stage the procedures or to perform them in a single session.

The rationale for a single-stage approach is that a median sternotomy affords simultaneous access to the anterior mediastinum and the left upper paratracheal compartment, enabling extended thymectomy and tumor enucleation within the same exposure. Although extended thymectomy alone is often performed via VATS or RATS, in this case the concomitant left RLN schwannoma required precise exposure for nerve-sparing enucleation; therefore, we elected median sternotomy. Using a shared operative field further promotes consistent identification of mediastinal neurovascular landmarks and obviates the scarring that might follow a prior operation, which could otherwise complicate subsequent dissection of the recurrent laryngeal nerve.

When the nerve course is anatomically clear and the lesion is suitable for intracapsular, nerve-sparing enucleation, intraoperative nerve monitoring need not be considered mandatory. While numerous studies have reported its usefulness in identifying the nerve and reducing traction-related injury [6], continuous vagus nerve or RLN stimulation–based monitoring has also been linked—albeit rarely—to clinically relevant adverse events such as RLN paralysis, bradycardia or even asystole, as well as device-related issues [7, 8]; thus, its application should be individualized rather than routine. In the present case, the monitoring system was prepared and kept on standby, with the intention of activating it if the RLN anatomy became unclear or if neural integrity was in question during dissection; however, the RLN course and fascicles were readily identified under direct vision, and stimulation was not activated. Practical maneuvers include maintaining a bloodless, magnified field, creating a longitudinal epineurial window away from visible fascicles, developing the intracapsular plane circumferentially, and progressively separating the encapsulated tumor from the nerve bed with fine blunt dissection while avoiding traction and thermal spread. It is important to confirm that macroscopic neural continuity is preserved while adhering to the above precautions. Our patient experienced no postoperative hoarseness, and laryngoscopy confirmed intact vocal fold mobility.

Perioperative management of MG and surveillance for RLN palsy require meticulous planning to avert respiratory complications. Preoperative evaluation should include assessment of MG functional status and laryngoscopic examination of vocal fold mobility. Postoperatively, if changes in voice or swallowing occur, laryngoscopic re-evaluation is mandatory. In addition, if an MG crisis develops, MG-directed rescue therapy—such as intravenous immunoglobulin or plasma exchange—must be immediately available [9, 10]. Notably, when RLN palsy and MG crisis occur concurrently, the respiratory condition can rapidly become critical, and tracheostomy may be required to secure the airway.

In this case, preoperative transesophageal fine-needle aspiration established the diagnosis of schwannoma, vocal fold mobility was intact, and MG was clinically stable. On that basis, we elected a single-stage strategy—extended thymectomy with enucleation of the RLN schwannoma—which was performed safely. However, when preoperative RLN palsy is present or MG control is suboptimal, postoperative respiratory deterioration must be anticipated, and adequate perioperative resources—including advanced airway support and rapid access to MG-directed rescue therapy—should be secured. As a single-case experience, our report is inherently limited in generalizability and follow-up duration; broader series are needed to define patient selection criteria and perioperative pathways more precisely.

## Conclusion

For carefully selected patients, a single-stage median sternotomy offers practical advantages and can achieve both disease control for MG and preservation of vocal fold function after RLN schwannoma enucleation, provided that perioperative plans explicitly address the compounded risks of airway compromise and myasthenic crisis.

## Data Availability

Data that support the findings of this study are not openly available due to [reasons of sensitivity e.g. human data] and are available from the corresponding author upon reasonable request [include information on the data’s location, e.g. in a controlled access repository where relevant].
